# Inhibition of PI3Kδ Differentially Regulates Poly I:C– and Human Metapneumovirus–Induced PD–L1 and PD–L2 Expression in Human Bronchial Epithelial Cells

**DOI:** 10.3389/fimmu.2021.767666

**Published:** 2021-11-25

**Authors:** Tomohiro Ogawa, Keiko Kan-o, Ayaka Shiota, Akitaka Fujita, Yumiko Ishii, Satoru Fukuyama, Koichiro Matsumoto

**Affiliations:** ^1^ Research Institute for Diseases of the Chest, Graduate School of Medical Sciences, Kyushu University, Fukuoka, Japan; ^2^ Department of Endoscopic Diagnostics and Therapeutics, Kyushu University Hospital, Fukuoka, Japan

**Keywords:** programmed cell death 1 ligand 1, programmed cell death 1 ligand 2, interferon, phosphoinositide 3-kinase delta, bronchial epithelial cells, human metapneumovirus

## Abstract

Bronchial epithelial cells are front sentinels eliciting innate and adaptive immunity to respiratory viral pathogens. Recognition of viral double-stranded RNA induces antiviral interferon (IFN) responses in bronchial epithelial cells. Co-inhibitory molecules programmed cell death 1 ligand 1 (PD-L1) and ligand 2 (PD-L2) were also induced on bronchial epithelial cells, which bind programmed cell death 1 on T cell and inhibit the function of virus-specific cytotoxic T lymphocyte. A previous study showed that antiviral type I IFN increased PD-L1 and PD-L2 expression in cultured melanoma cells. However, it remains unknown whether antiviral IFNs affect PD-L1 and PD-L2 expression in bronchial epithelial cells. In addition, we previously reported that inhibition of PI3Kδ signaling enhanced antiviral IFN responses in human primary bronchial epithelial cells (PBECs). Here we assessed the effect of exogenous IFNs or a selective PI3Kδ inhibitor IC87114 on PD-L1 and PD-L2 in PBECs stimulated with a synthetic double-stranded RNA poly I:C or human metapneumovirus. Treatment with IFNβ or IFNλ increased PD-L1 and PD-L2, and IFNβ or IFNλ treatment plus poly I:C further increased both expressions. Treatment with IC87114 or transfection with siRNA targeting PI3K p110δ enhanced poly I:C–induced gene and protein expression of PD-L2, whereas IC87114 suppressed poly I:C–induced PD-L1. IC87114 enhanced poly I:C–induced gene expression of IFNβ, IFNλ, and IFN-regulated genes *via* increased TBK1 and IRF3 phosphorylation. Transfection with siIRF3 counteracted the enhancement of poly I:C–induced PD-L2 by IC87114, whereas IC87114 suppressed poly I:C–induced PD-L1 regardless of transfection with siNC or siIRF3. Similar effects of IC87114 on PD-L1 and PD-L2 expression were observed in human metapneumovirus–infected PBECs. We showed for the first time that type I and type III IFNs induced the expression of PD-L1 and PD-L2 in PBECs. Our findings suggest that during viral infections, inhibition of PI3Kδ differentially regulates PD-L1 and PD-L2 expression in bronchial epithelial cells.

## Introduction

Programmed cell death 1 ligand 1 (PD-L1) and ligand 2 (PD-L2) are ligands of the programmed cell death 1 (PD-1) co-inhibitory receptor on T cells. The PD-1/PD-L1 axis is a negative modulatory signaling pathway for the activation of T cells, and recent experimental evidence has shown that the PD-1/PD-L1 axis also regulates antiviral immune responses (1–3). In respiratory virus infections, such as human metapneumovirus (hMPV) or influenza virus infection, inhibition of the PD-1/PD-L1 pathway restored virus-specific CD8^+^ T cell activity and resulted in faster virus clearance in lungs, which suggests that PD-L1 upregulation on infected cells is responsible for CD8^+^ T cell exhaustion (2, 4, 5). Although studies have shown that the expression of PD-L2 is induced in bronchial epithelial cells following viral infection such as respiratory syncytial virus (6), the precise role of PD-L2 in the antiviral immune response remains unknown.

Respiratory virus infections exacerbate chronic lung diseases such as asthma and chronic obstructive pulmonary disease (COPD). Bronchial epithelial cells are the primary target of infections by viruses such as rhinovirus, respiratory syncytial virus, influenza virus, and hMPV. These single-stranded RNA viruses generate double-stranded RNA (dsRNA) or a panhandle-like dsRNA structure in infected host cells during replication. Viral dsRNA is recognized by pattern-recognition receptors (PRRs), such as toll-like receptor (TLR) 3 on endosomes and cytosolic RNA helicase retinoic acid-inducible gene-I or melanoma differentiation-associated gene 5, leading to the secretion of antiviral interferons (IFNs) and pro-inflammatory cytokines and chemokines (7–9). Type I IFNs (IFNα and IFNβ) and type III IFNs (IFNλ) secreted from infected bronchial epithelial cells stimulate neighboring cells through IFN receptors to express IFN-regulated genes (IRGs), inducing an antiviral state (10, 11). We previously showed that viral infection or stimulation with a synthetic dsRNA analog, polyinosinic-polycytidylic acid (poly I:C), increased PD-L1 and PD-L2 expression on human bronchial epithelial cells, and the NF-κB pathway plays an essential role in poly I:C–induced upregulation of PD-L1 (12–14). Recent studies showed that exogenous type I IFN induces not only PD-L1 expression in endothelial cells, monocytes, and dendritic cells but also PD-L2 expression in cultured melanoma cells (15–17). While type I and III IFNs are induced as the first line response against respiratory viral infection, the effect of antiviral IFNs on the expression of PD-L1 and PD-L2 in bronchial epithelial cells has not been investigated.

Phosphoinositide 3-kinases (PI3Ks) regulate various biological functions including cell proliferation, differentiation, and migration *via* several downstream molecules such as v-akt murine thymoma viral oncogene homolog (Akt) and the mechanistic target of rapamycin (mTOR). PI3Kδ is a class IA PI3K and predominantly expressed in cells of the myeloid and lymphoid lineages (18). We recently reported that inhibition of PI3Kδ signaling enhanced poly I:C–induced antiviral IFN responses in human primary bronchial epithelial cells (PBECs), which suggests that PI3Kδ may negatively regulate the antiviral IFN response (14). We have also shown that a selective PI3Kδ inhibitor attenuated poly I:C–induced PD-L1 expression by inhibiting translational induction of PD-L1 *via* the Akt/mTOR pathway (14). However, the association between PI3Kδ signaling and poly I:C–induced expressions of PD-L2 on PBECs remains unknown.

In this study, we assessed whether exogenous IFNs induce the expression of PD-L1 and PD-L2 using PBECs and treatment with IFN plus poly I:C further enhances PD-L1 and PD-L2. We also evaluated whether inhibition of PI3Kδ signaling affects the poly I:C–induced activation of downstream transcription factors of PRRs, antiviral IFN responses, and expression of PD-L1 and PD-L2 in healthy PBECs and PBECs from asthma patients and COPD patients. Finally, we analyzed the effect of the selective PI3Kδ inhibitor on hMPV-induced PD-L1 and PD-L2 expressions on PBECs.

## Materials and Methods

### Culture and Treatment of PBECs

PBECs were collected and cultured according to our previously described protocol (14). Briefly, PBECs were obtained during routine fibreoptic bronchoscopy from never-smoker patients with normal lung function and pulmonary nodules by performing bronchial brushings from healthy lobes without a pulmonary nodule (healthy PBECs). All bronchial brushings were obtained from the same anatomical region (bronchial generations 4–7). PBECs were also collected from patients with asthma and patients with COPD for use in some experiments. The characteristics of patients with asthma or COPD at bronchoscopy are shown in **Table S1**. Unless otherwise stated, healthy PBECs were used in the experiments. The study protocol was approved by the Kyushu University Institutional Review Board for Clinical Research (29-170). All subjects provided written informed consent in accordance with the principles laid out in the Declaration of Helsinki.

Cells were cultured in flasks coated with collagen (Cell Applications, Inc., San Diego, CA, USA) containing supplemented bronchial epithelial growth medium (BEGM; Lonza, Basel, Swiss) at 37°C in 5% CO_2_ and used within four passages. PBECs (approximately 80% confluency) were pretreated with 250 U/ml human IFNβ1a (PBL Assay Science, Piscataway, NJ, USA), 250 U/ml human IFNλ1 (PBL Assay Science), 10 μM IC87114 (BioVision, Milpitas, CA, USA), 10 μM AS604850 (FUJIFILM Wako Pure Chemical Corporation, Osaka, Japan) or vehicle for 1 h and then stimulated by the addition of 1 μg/mL poly I:C (Innaxon, Tewkesbury, United Kingdom) to the culture medium. In virus infection experiments, 10 μM IC87114 was added to cells for 1 h prior to hMPV infection, and IC87114 was maintained in medium after infection. PBECs were cultured in BEGM without hydrocortisone for at least 24 h prior to stimulation or infection.

### Flow Cytometry

PBECs were cultured to semi-confluence in 12-well plates. Cells were incubated in 100 μL of PBS containing 0.5% BSA containing PE anti-human PD-L1 (clone: MIH1; Invitrogen, Carlsbad, CA, USA) or PE anti-human PD-L2 (clone: MIH18; Invitrogen) at room temperature for 30 min in initial experiments. In some experiments, cells were incubated in 100 μL of PBS with 0.5% BSA containing APC anti-human PD-L1 (clone: MIH1; Invitrogen) and PE anti-human PD-L2 (clone: MIH18; Invitrogen) at room temperature for 30 min. Cells were thoroughly washed and analyzed using a BD FACSVerse flow cytometer with FACSuite software (Becton Dickinson, Franklin Lakes, NJ, USA). Ten thousand events were acquired in list mode with debris excluded by the forward scatter threshold.

For the analysis of cell viability, resuspended lung cells or PBECs were incubated with propidium iodide (PI), and non-viable cells (stained with PI) were counted in a flow cytometer with debris excluded by the forward scatter threshold.

### Transfection of Small Interfering RNA

PBECs cultured in 6-well plates (60%–80% confluence) were transiently transfected with 10 nM IFN regulatory factor 3 (IRF3) siRNA (Silencer^®^ Select Validated siRNA, s7507; Ambion, Life Technologies, Carlsbad, CA, USA), 10 nM PIK3CD siRNA (Silencer^®^ Select Validated siRNA, s10529; Ambion, Life Technologies) or 10 nM negative control (NC) siRNA (Silencer^®^ Select Negative Control siRNA, 4390843; Ambion, Life Technologies) using Lipofectamine^®^ RNAiMAX Reagent (Invitrogen) according to the manufacturer’s instructions. Lipofectamine^®^ RNAiMAX Reagent or siRNA were diluted in BEGM without hydrocortisone and antibiotics. Cells were used for experiments at 48 h after transfection.

### Western Blotting

PBECs cultured to semi-confluence in 6-well plates were lysed using Pierce^®^ RIPA Buffer (Thermo Fisher Scientific, San Diego, CA, USA) according to the manufacturer’s instructions. Protein samples (4 µg) were denatured, separated by SDS-PAGE, and transferred to a polyvinylidene difluoride membrane. Membranes were blocked with TBS-Tween (10 mM Tris, 150 mM NaCl, 0.05% Tween 20, pH 8.0) containing 5% skimmed milk for 1 h at room temperature. The membrane was then incubated with anti-PD-L1 (Cell Signaling Technology), anti-PD-L2 (Cell Signaling Technology), anti-the TANK-binding kinase-1 (TBK1) (Cell Signaling Technology, Beverly, MA, USA), anti-phospho-TBK1 (Ser172, Cell Signaling Technology), anti-IRF3 (Cell Signaling Technology), anti-phospho-IRF3 (Ser386, Cell Signaling Technology), anti-PI3 Kinase p110δ (Cell Signaling Technology) or anti-β-actin (Santa Cruz Biotechnology, Dallas, TX, USA) antibodies at 4°C overnight. Membranes were washed three times with TBS-Tween and then incubated with a horseradish peroxidase-conjugated secondary antibody for 60 min at room temperature. Membranes were washed three times with TBS-Tween, and specific bands were visualized using ImmunoStar^®^ LD (Wako, Osaka, Japan) according to the manufacturer’s instructions. All blots were imaged using the ChemiDoc™ XRS+ system (Bio-Rad Laboratories, Inc., Hercules, CA, USA). Densitometric analysis of band intensities was performed using Image J.

### Quantitative Reverse-Transcription PCR

Total RNA was isolated from PBECs using TRI Reagent (Molecular Research Centre, Inc., Cincinnati, OH, USA). Reverse transcription was performed using Multiscribe Reverse Transcriptase (Invitrogen). Real-time quantitative RT-PCR analyses were performed once per sample using Fast Start SYBR Green Master (Roche, Mannheim, Germany) and a Thermal Cycler Dice Real Time System II (Takara, Shiga, Japan). The mRNA expression levels were calculated from the threshold cycle according to the ΔΔCt method. Target gene expression levels were normalized to the expression of 18S rRNA. Primer sequences are provided in **Table S2**.

### Virus Infection of Cultured Cells

The CAN97-83 strain of hMPV was used; hMPV was propagated in Vero E6 cells (CRL-1586; ATCC, Manassas, VA, USA) and virus titer was determined by TCID_50_ assay. In brief, virus was prepared in serum-free MEM (Gibco, Thermo Fisher Scientific, San Diego, CA, USA) containing 5 μg/ml trypsin (Gibco) by infecting cells in a 10-cm culture dish at a multiplicity of infection (MOI) of 0.05. After absorption for 2 h at 37°C, serum-free MEM containing 5 μg/ml trypsin was added. Serum-free medium containing 5 μg/ml trypsin was changed every other day during incubation and cells were incubated until a 70%–90% cytopathic effect was observed, usually within 7 days. Cells and culture supernatant were　collected and virus was obtained by freeze-thawing the cells. This strain of hMPV has been used in other studies (19), and we did not attempt further purification to avoid altering key properties of the virus (20).

Virus was quantified by immunostaining assay. Briefly, semi-confluent Vero E6 monolayers in a 24-well plate were infected with 200 μl of 10-fold serial dilutions. Trypsin (5 μg/ml) was added in serum-free MEM during infection. After absorption for 2 h at 37°C, 200 μl of MEM containing 5% FBS was added. After 6 days of cell culture, infected wells were identified by immunostaining with mouse anti-hMPV monoclonal primary antibody (MAB8510; EMD Millipore, Temecula, CA, USA) and horseradish peroxidase-labeled goat anti-mouse secondary antibody (Abcam, Cambridge, UK). Cells were visualized using DAB peroxidase substrate (Vector Laboratories, Inc., Burlingame, CA, USA) and the TCID_50_/ml was determined using standard methods.

Control experiments using UV-inactivated hMPV were included to confirm that responses were a result of hMPV infection and not from factors remaining in the culture media following hMPV propagation. To UV-irradiate virus, hMPV was exposed to a short wavelength (254 nm) UV lamp at a distance of 5 cm for 15 min. PBECs (approximately 80% confluency) were washed with PBS and infected with hMPV in BEGM without hydrocortisone for 1 h at a MOI of 0.1. Cell monolayers were washed and then incubated in BEGM without hydrocortisone for 24–72 h. Standard biosecurity and institutional safety procedures were followed during this study.

### Statistical Analyses

Unless otherwise stated, data were expressed as means ± standard error (SEM). The Mann–Whitney U-test was used for comparisons between two groups. Comparisons of three or more groups were conducted using one-way analysis of variance (ANOVA) or two-way ANOVA followed by Tukey’s multiple comparisons test. Correlations were examined using Spearman correlation. All statistical analyses were conducted using GraphPad Prism 8 software (GraphPad Software, San Francisco, CA, USA). Differences were considered statistically significant at *p*<0.05.

## Results

### Antiviral IFNs Increase the Expression of PD-L1 and PD-L2 on PBECs

**Figure 1:**
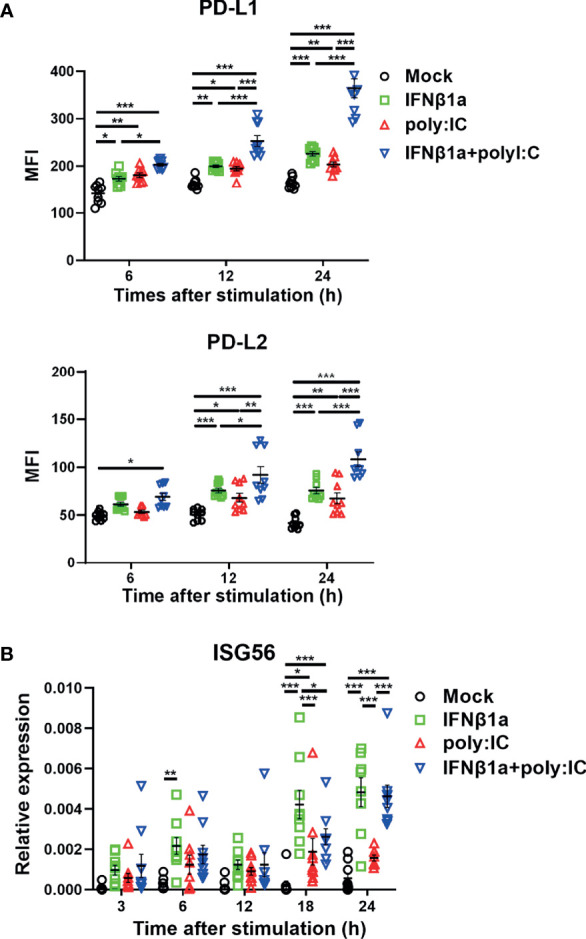
Antiviral interferon increases the expression of PD-L1 and PD-L2 on PBECs. PBECs were pretreated with 250 U/ml IFNβ1a or vehicle for 1 h and then stimulated with 1 μg/mL poly I:C. **(A)** PD-L1 expression and PD-L2 expression were analyzed at the indicated times following stimulation using flow cytometry. **(B)** ISG56 gene expressions were measured at the indicated times by real-time quantitative reverse-transcription PCR and normalized to that of 18S rRNA. Data represent means ± SEM (n=9 per group) and were pooled from three independent donors with three replicates. *p<0.05, **p<0.01, ***p<0.001 by two-way ANOVA. MFI, mean fluorescence intensity.

Stimulation with IFNβ1a alone or poly I:C alone increased the expression of PD-L1 and PD-L2 on PBECs at 6 and 12 h following stimulation, respectively (**Figure 1A**). The combination treatment of IFNβ1a plus poly I:C upregulated the expression of PD-L1 and PD-L2 to higher levels compared with stimulation with IFNβ1a or poly I:C alone. Stimulation with IFNβ1a alone or the combination treatment of IFNβ1a plus poly I:C induced higher gene expression levels of IFN-stimulated gene (*ISG*) *56* than stimulation with poly I:C alone (**Figure 1B**). Similarly, IFNλ1 or poly I:C alone increased the expression of PD-L1 and PD-L2 on PBECs at 24 h after stimulation, and further upregulation of PD-L1 and PD-L2 was observed after the combination treatment of IFNλ1 plus poly I:C (**Figure S1A**). The cell viability of PBECs at 24 h after stimulation was not affected by treatment with IFNβ1a or IFNλ1 and/or poly I:C (**Figure S1B**).

### Antiviral IFN Induces the Gene and Protein Expression of PD-L1 and PD-L2 in PBECs

**Figure 2:**
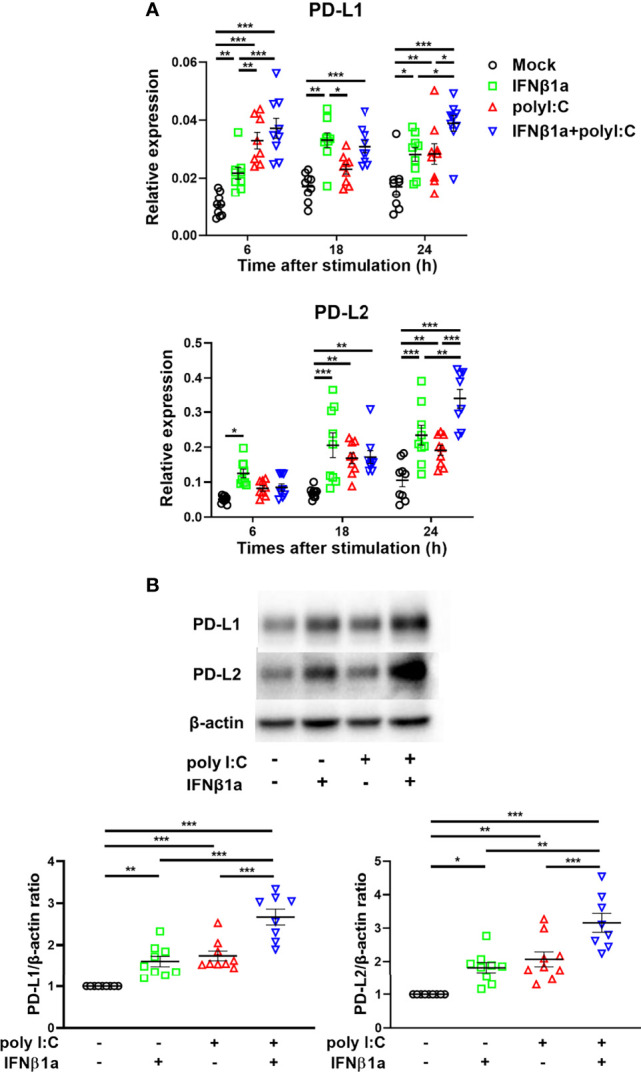
Antiviral interferon induces gene and protein expressions of PD-L1 and PD-L2 in PBECs. PBECs were pretreated with 250 U/ml IFNβ1a or vehicle for 1 h and then stimulated with 1 μg/mL poly I:C. **(A)** PD-L1 and PD-L2 gene expressions were measured at the indicated times by real-time quantitative reverse-transcription PCR and normalized to that of 18S rRNA. **(B)** Representative western blots showing PD-L1 and PD-L2 in PBECs 24 h following stimulation. Band intensity was quantitated using densitometry. Data represent means ± SEM (n=9 per group) and were pooled from three independent donors with three replicates. *p<0.05, **p<0.01, ***p<0.001 by one- o two-way ANOVA.

Next, we assessed the effect of treatment with IFNβ1a and/or poly I:C on gene expression of *PD-L1* and *PD-L2*. Treatment with IFNβ1a alone or poly I:C alone induced *PD-L1* and *PD-L2* gene expression 6 h following stimulation (**Figure 2A**). The combination treatment of IFNβ1a plus poly I:C further increased gene expression levels of *PD-L1* and *PD-L2* compared to treatment with IFNβ1a or poly I:C alone. IFNβ1a alone or poly I:C alone also increased the protein levels of PD-L1 and PD-L2 at 24 h following stimulation, and combination treatment enhanced both protein levels (**Figure 2B**).

### A PI3Kδ Inhibitor Enhances Poly I:C–Induced PD-L2 Expression on PBECs, Whereas It Suppresses Poly I:C–Induced PD-L1 Expression

**Figure 3:**
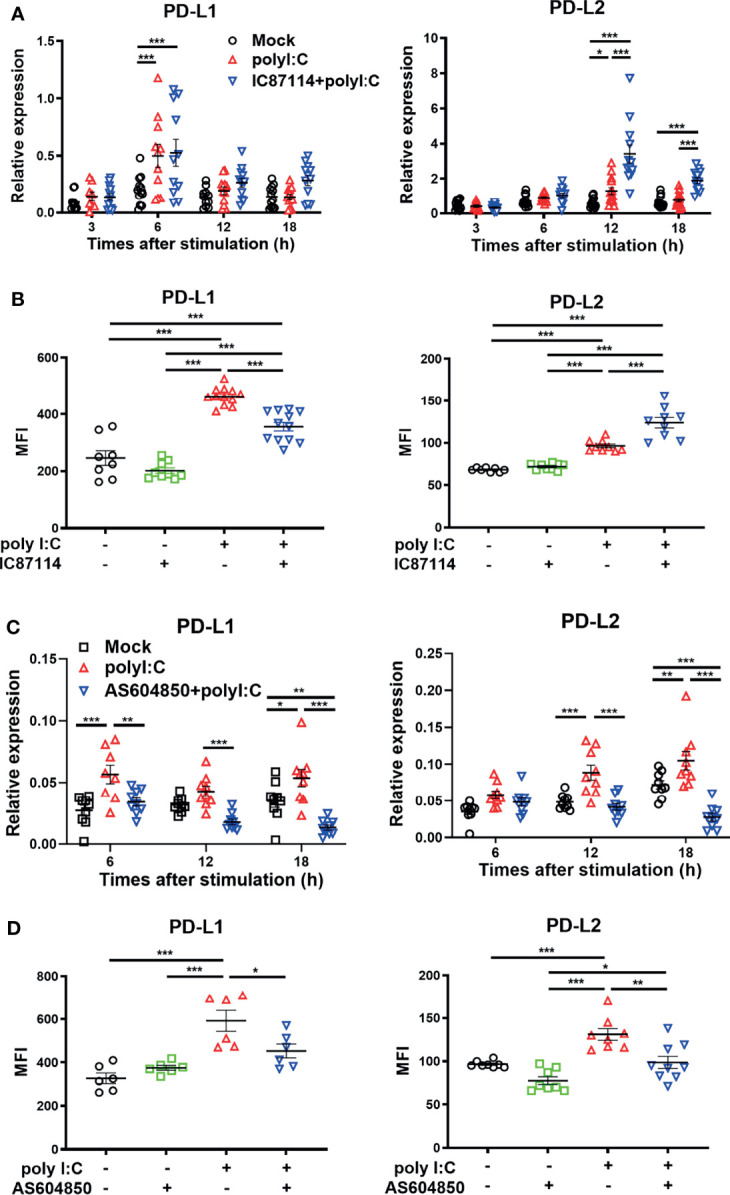
A PI3Kδ inhibitor enhances poly I:C–induced PD-L2 expression on PBECs and suppresses poly I:C–induced PD-L1 expression. PBECs were pretreated with 10 μM the PI3Kδ inhibitor IC87114 (A, B), 10 μM the PI3Kγ inhibitor AS604850 **(C, D)**, or vehicle for 1 h and then stimulated with 1 μg/mL poly I:C for the indicated times. **(A, C)** PD-L1 and PD-L2 gene expressions were measured by real-time quantitative reverse-transcription PCR and normalized to that of 18S rRNA. **(B, D)** PD-L1 expression and PD-L2 expression were analyzed 24 h following stimulation using flow cytometry. Data represent means ± SEM (n=6–12 per group) and were pooled from a minimum of two independent donors with three replicates. *p<0.05, **p<0.01, ***p<0.001 by one- or two-way ANOVA as appropriate. MFI, mean fluorescence intensity.

We previously reported that the selective PI3Kδ inhibitor IC87114 attenuated poly I:C–induced PD-L1 expression on bronchial epithelial cells *via* inhibition of the Akt/mTOR signaling pathway (14). However, it has been unclear whether IC87114 affects poly I:C–induced PD-L2 expression. Here we analyzed the effects of IC87114 on the expression of PD-L1 and PD-L2 in PBECs. Although IC871114 did not affect poly I:C–induced gene expression levels of *PD-L1*, IC87114 markedly increased poly I:C–induced gene expression levels of *PD-L2* at 12 h and 18 h following stimulation with poly I:C (**Figure 3A**). Consistent with our previous report, IC87114 suppressed poly I:C–induced PD-L1 expression at 24 h after poly I:C stimulation (**Figure 3B**). In contrast, IC87114 significantly enhanced poly I:C–induced PD-L2 expression. However, the combination treatment of IC87114 plus IFNβ1a did not additively enhanced gene expression levels of PD-L1 and PD-L2 (**Figure S2**). PI3Kγ knockout mice were reported to be highly susceptible to lethality following infection with influenza A virus due to impaired antiviral IFNs responses (21). Hence, we also evaluated the effects of the selective PI3Kγ inhibitor AS604850 on the expression of PD-L1 and PD-L2. Unlike IC87114, AS604850 suppressed poly I:C–mediated gene and protein induction of both PD-L1 and PD-L2 expressions (**Figures 3C, D**). AS604850 also decreased poly I:C-induced gene expression levels of *ISG56* (**Figure S3A**). Treatment with IC87114 or AS604850 with or without poly I:C did not affect the cell viability of PBECs (**Figure S3B**). These results indicate that inhibition of PI3Kδ signaling enhanced poly I:C–induced PD-L2 expression on PBECs, while inhibition of PI3Kγ signaling did not have this effect.

### siRNA Knockdown of *PIK3CD* Enhances Poly I:C–Induced Upregulation of PD-L2 Expression on PBECs

**Figure 4:**
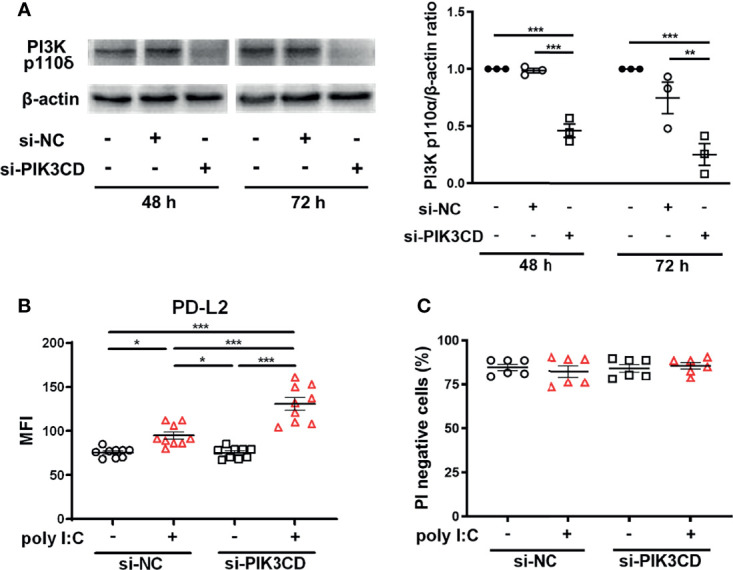
siRNA knockdown of *PIK3CD* enhances poly I:C–induced upregulation of PD-L2 expression on PBECs. **(A)** Representative western blots showing PI3K p110δ in PBECs transfected with 10 nM PIK3CD siRNA or 10 nM negative control (NC) siRNA for 48 h or 72 h. Band intensity was quantitated using densitometry. Data represent means ± SEM (n=3 per group) of two replicates from two independent donors. **(B, C)** PBECs were transfected with 10 nM PIK3CD siRNA or 10 nM NC siRNA for 48 h and then stimulated with 1 μg/mL poly I:C or vehicle for 24 h. PD-L2 expression or PI-negative viable cells were analyzed using flow cytometry. Data represent means ± SEM (n=6–9 per group) were pooled from a minimum of two independent donors with three replicates. *p<0.05, **p<0.01, ***p<0.001 by one- or two-way ANOVA as appropriate. MFI, mean fluorescence intensity.

To confirm that inhibition of PI3Kδ signaling enhanced poly I:C–induced upregulation of PD-L2, we examined the effect of knockdown of the *PIK3CD* gene, which encodes PI3K p110δ, in PBECs. PI3K p110δ expression was suppressed in PBECs transfected with siPIK3CD at 48 h and 72 h following transfection compared with cells transfected with si-negative control (NC) (**Figure 4A**). PBECs transfected with siPIK3CD or siNC for 48 h were stimulated with poly I:C for 24 h, and PD-L2 expression was analyzed. Untreated cells transfected with siPIK3CD showed no changes in PD-L2 expression (**Figure 4B**). However, poly I:C–induced PD-L2 expression was enhanced on PBECs transfected with siPIK3CD compared with cells transfected with siNC (**Figure 4B**). Cell viability of PBECs was not affected by siRNA knockdown of PIK3CD and stimulation of poly I:C (**Figure 4C**).

### A PI3Kδ Inhibitor Enhances Poly I:C–Induced Antiviral IFN Responses by Promoting TBK1/IRF3 Phosphorylation in PBECs

**Figure 5:**
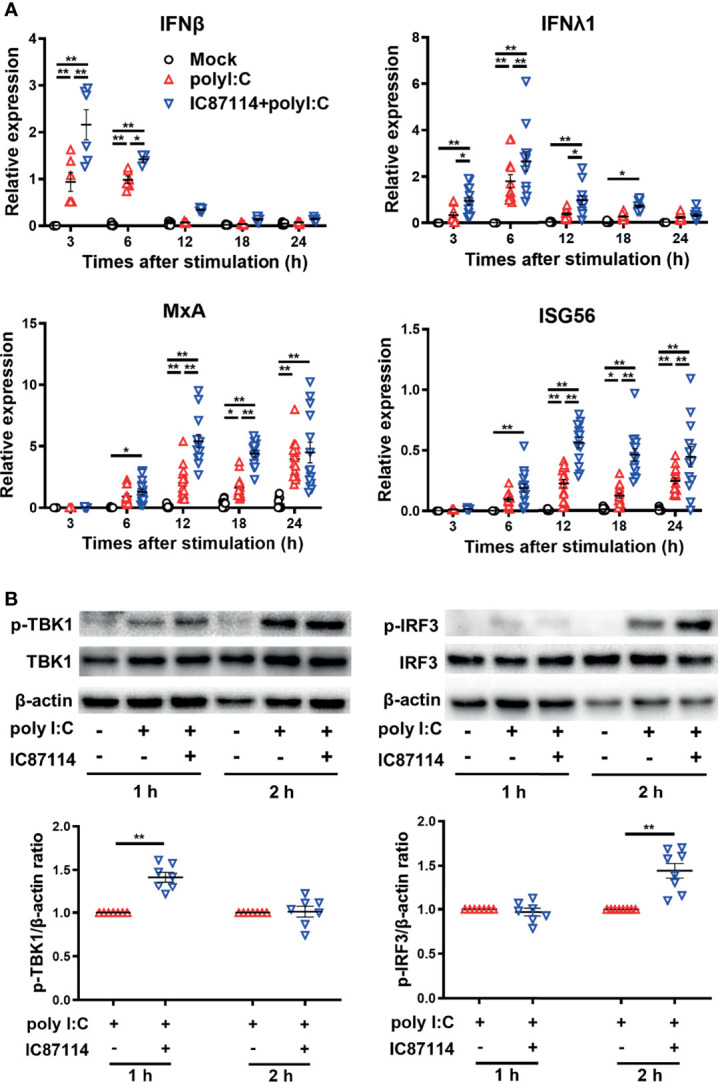
A PI3Kδ inhibitor enhances poly I:C–induced antiviral IFN responses by promoting TBK1/IRF3 phosphorylation in PBECs. PBECs were pretreated with 10 μM IC87114 or vehicle for 1 h and then stimulated with 1 μg/mL poly I:C for the indicated times. **(A)** IFNβ, IFNλ1, MxA, and ISG56 gene expressions were measured by real-time quantitative reverse-transcription PCR and normalized to that of 18S rRNA. Data represent means ± SEM (n=6–12 per group) and pooled from a minimum of two independent donors with three replicates. **(B)** Representative western blots showing phosphorylated TBK1, total TBK1, phosphorylated IRF3, and total IRF3 in PBECs. Band intensity was quantitated using densitometry. Data represent means ± SEM (n=7 per group) were pooled from four independent donors with two replicates. *p<0.05, **p<0.001 by two-way ANOVA.

We previously reported that IC87114 enhanced poly I:C–induced protein levels of IFNβ and IFNλ in bronchial epithelial cell culture supernatants (14). To investigate the mechanism of enhanced gene and protein expression of PD-L2 by IC87114 plus poly I:C, we examined the effects of IC87114 on the gene expression levels of poly I:C–induced antiviral IFNs and IRGs in PBECs. Stimulation with poly I:C increased gene expression levels of *IFNβ* and *IFNλ* at 3 h and 6 h following stimulation, respectively, as well as IRGs such as *MxA* and *ISG56* at later time points (**Figure 5A**). Treatment with IC87114 together with poly I:C significantly enhanced gene expression levels of IFNs and IRGs compared with levels in cells treated with poly I:C alone.

Activation of TBK1/IRF3 pathway is essential to elicit antiviral IFN responses after the binding of viral dsRNA to toll-like receptor 3 (22). Thus, we examined the effects of IC87114 on TBK1 and IRF3 phosphorylation in PBECs by western blot analysis. The combined treatment of IC87114 plus poly I:C significantly increased the levels of phosphorylated TBK1 and IRF3 at 1 h and 2 h following stimulation, respectively, compared with levels in cells treated with poly I:C alone (**Figure 5B**). Total TBK1 and IRF3 levels were not affected by the combined treatment of IC87114 and poly I:C stimulation (**Figure S4**).

### siRNA Knockdown of *IRF3* Counteracts the Enhancement of Poly I:C–Induced PD-L2 Expression by a PI3Kδ Inhibitor

**Figure 6:**
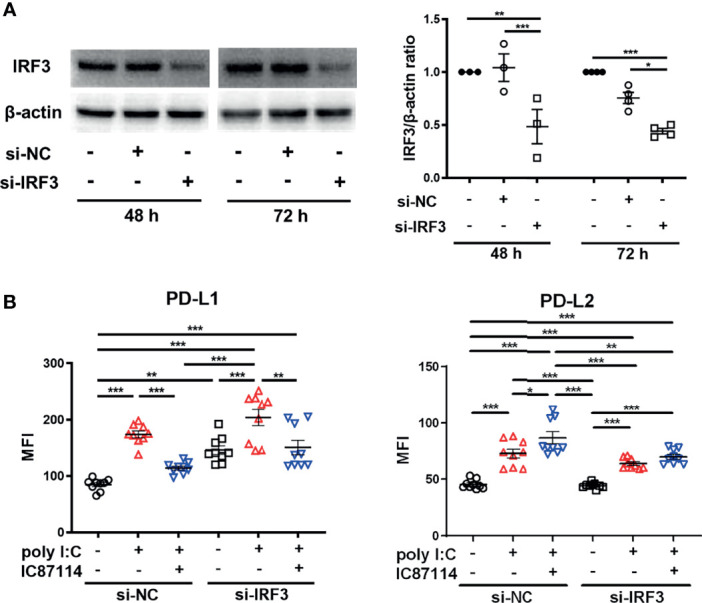
siRNA knockdown of *IRF3* counteracts enhancement of poly I:C–induced PD-L2 expression by a PI3Kδ inhibitor. **(A)** Representative western blots of IRF3 in PBECs transfected with 10 nM IRF3 siRNA or 10 nM negative control (NC) siRNA for 48 h or 72 h. Band intensity was quantitated using densitometry. Data represent means ± SEM (n=3–4 per group) and pooled from two independent donors with two replicates. **(B)** PBECs were transfected with 10 nM IRF3 siRNA or 10 nM NC siRNA for 48 h. Transfected cells were pretreated with 10 μM IC87114 or vehicle for 1 h followed by stimulation with 1 μg/mL poly I:C for 24 h PD-L1 and PD-L2 expressions on PBECs were analyzed using flow cytometry. Data represent means ± SEM (n=9 per group) and pooled from three independent donors with three replicates. *p<0.05, **p<0.01, ***p<0.001 by one-way ANOVA. MFI, mean fluorescence intensity.

Next, we assessed the effects of knockdown of the *IRF3* gene, which encodes a key transcription factor in the poly I:C–induced antiviral IFNs response, on the expression of PD-L1 and PD-L2 in PBECs stimulated with poly I:C with or without IC87114. We confirmed decreased protein levels of IRF3 in PBECs at 48 h and 72 h following siIRF3 transfection (**Figure 6A**). PBECs transfected with siIRF3 or siNC for 48 h were stimulated with poly I:C alone or IC87114 plus poly I:C, and the expressions of PD-L1 and PD-L2 were analyzed 24 h following stimulation. We previously showed that the NF-κB pathway plays an essential role in poly I:C–induced upregulation of PD-L1 (23). Knockdown of the *IRF3* gene did not attenuate poly I:C–induced PD-L1 and PD-L2 expression (**Figure 6B**). Regardless of transfection with siNC or siIRF3, IC87114 suppressed poly I:C–induced PD-L1 expression. Furthermore, the enhancement of poly I:C–induced PD-L2 expression by IC87114 was counteracted in PBECs transfected with siIRF3. Cell viability was not affected by stimulation of poly I:C alone or combined with IC87114 after siNC or siIRF3 transfection (**Figure S5**).

### The Effects of a PI3Kδ Inhibitor on Poly I:C–Induced PD-L1 And PD-L2 Expression in PBECs From Patients With Asthma or COPD

**Figure 7:**
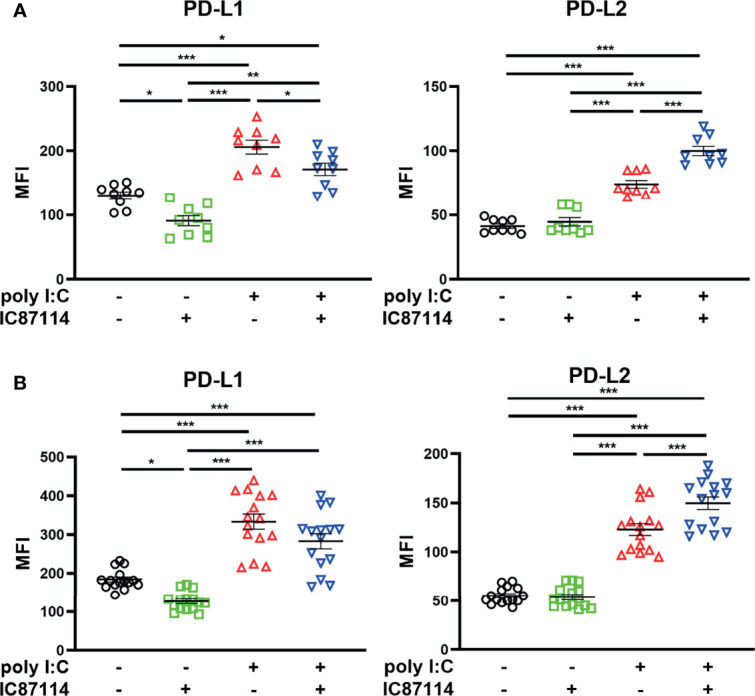
The effects of a PI3Kδ inhibitor on poly I:C–induced PD-L1 and PD-L2 expression on PBECs from patients with asthma or COPD. PBECs from patients with asthma **(A)** or COPD **(B)** were pretreated with 10 μM IC87114 or vehicle for 1 h and then stimulated with 1 μg/mL poly I:C for 24 h. PD-L1 and PD-L2 expression on PBECs was analyzed using flow cytometry. Data represent means ± SEM (n=9–12 per group) and pooled from a minimum of three independent donors with three replicates. *p<0.05, **p<0.01, ***p<0.001 by one-way ANOVA. MFI, mean fluorescence intensity.

The effects of IC87114 on poly I:C–induced PD-L1 and PD-L2 expressions were assessed using PBECs collected from patients with asthma or COPD. Similar to observations in healthy PBECs, treatment with IC87114 suppressed poly I:C–induced PD-L1 and increased poly I:C–induced PD-L2 in PBECs from patients with asthma (**Figure 7A**). In PBECs from patients with COPD, treatment with IC87114 increased poly I:C–induced PD-L2, but it did not affect poly I:C–induced PD-L1 (**Figure 7B**).

### A PI3Kδ Inhibitor Alters hMPV–Induced Gene and Protein Expression Levels of PD-L1 and PD-L2 in PBECs

**Figure 8:**
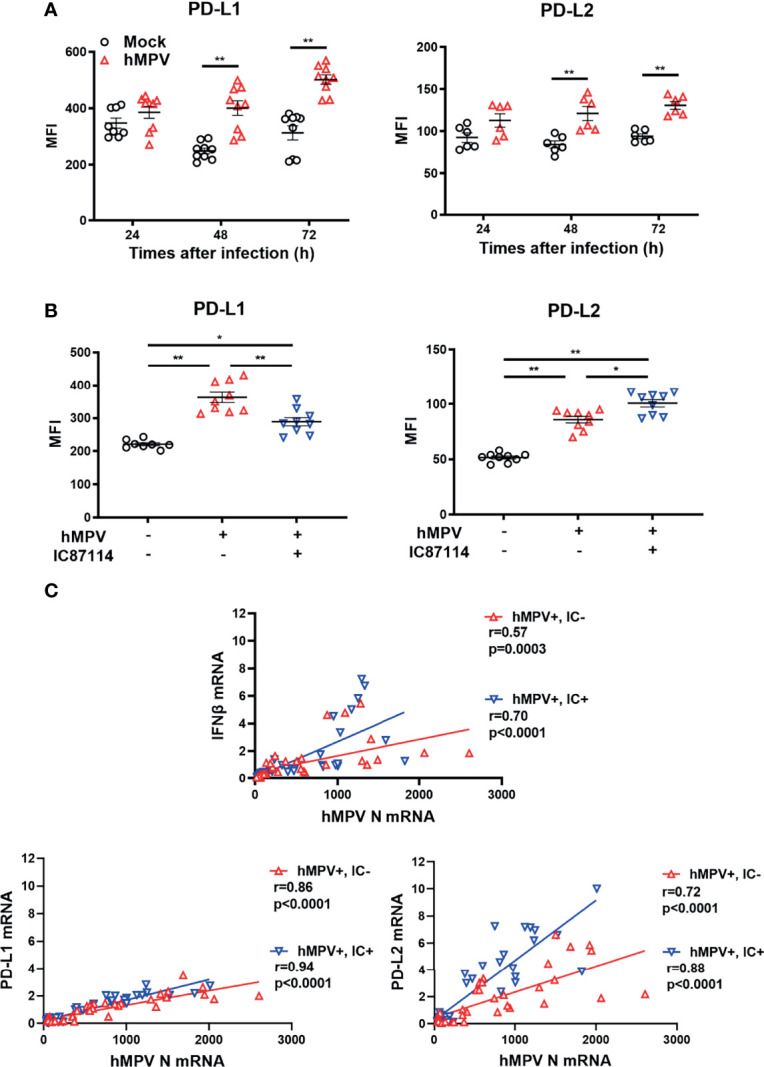
A PI3Kδ inhibitor alters hMPV-induced gene and protein expression levels of PD-L1 and PD-L2 in PBECs. **(A)** PBECs were infected with hMPV (MOI 0.1), and PD-L1 and PD-L2 expressions were analyzed at the indicated times using flow cytometry. **(B, C)** IC87114 (10 μM) or vehicle was added prior to and after hMPV infection (MOI 0.1). **(B)** PD-L1 and PD-L2 expressions were analyzed at 48 h post-infection using flow cytometry. MFI, mean fluorescence intensity. **(C)** Correlation between the gene expression of hMPV nucleocapsid protein (hMPV N) and IFNβ, PD-L1, and PD-L2 mRNAs. Cell lysates for RNA extraction were collected at 24, 36, and 48 h post-infection. Gene expressions were measured by real-time quantitative reverse-transcription PCR and normalized to that of 18S rRNA. Data represent means ± SEM (n=6–12 per group) and pooled from a minimum of two independent donors with three replicates. *p<0.01, **p<0.001 by one- or two-way ANOVA and Spearman correlation as appropriate. MFI, mean fluorescence intensity.

Finally, we investigated whether IC87114 affects virus-induced PD-L1 and PD-L2 expression in PBECs. Infection of PBECs with hMPV at a MOI of 0.1 induced the expression of PD-L1 and PD-L2 at 48 h and 72 h post-infection, and UV-irradiated hMPV did not increase PD-L1 or PD-L2 expression (**Figure 8A**, **S6**). Similar to observations in PBECs treated with IC87114 plus poly I:C, hMPV-induced PD-L1 expression was suppressed in PBECs treated with IC87114 at 48 h post-infection, while IC87114 enhanced hMPV-induced PD-L2 expression (**Figure 8B**). There was a linear association between the gene expression of hMPV nucleocapsid protein (*hMPV N*) and hMPV-induced *IFNβ*, *PD-L1*, and *PD-L2* in cells treated with or without IC87114 (**Figure 8C**). Treatment with IC87114 changed the slope of a regression line of *IFNβ* and *PD-L2* larger, which indicates more efficient induction of *IFNβ* and *PD-L2* gene responsive to hMPV infection in cells treated with IC87114.

## Discussion

**Figure 9:**
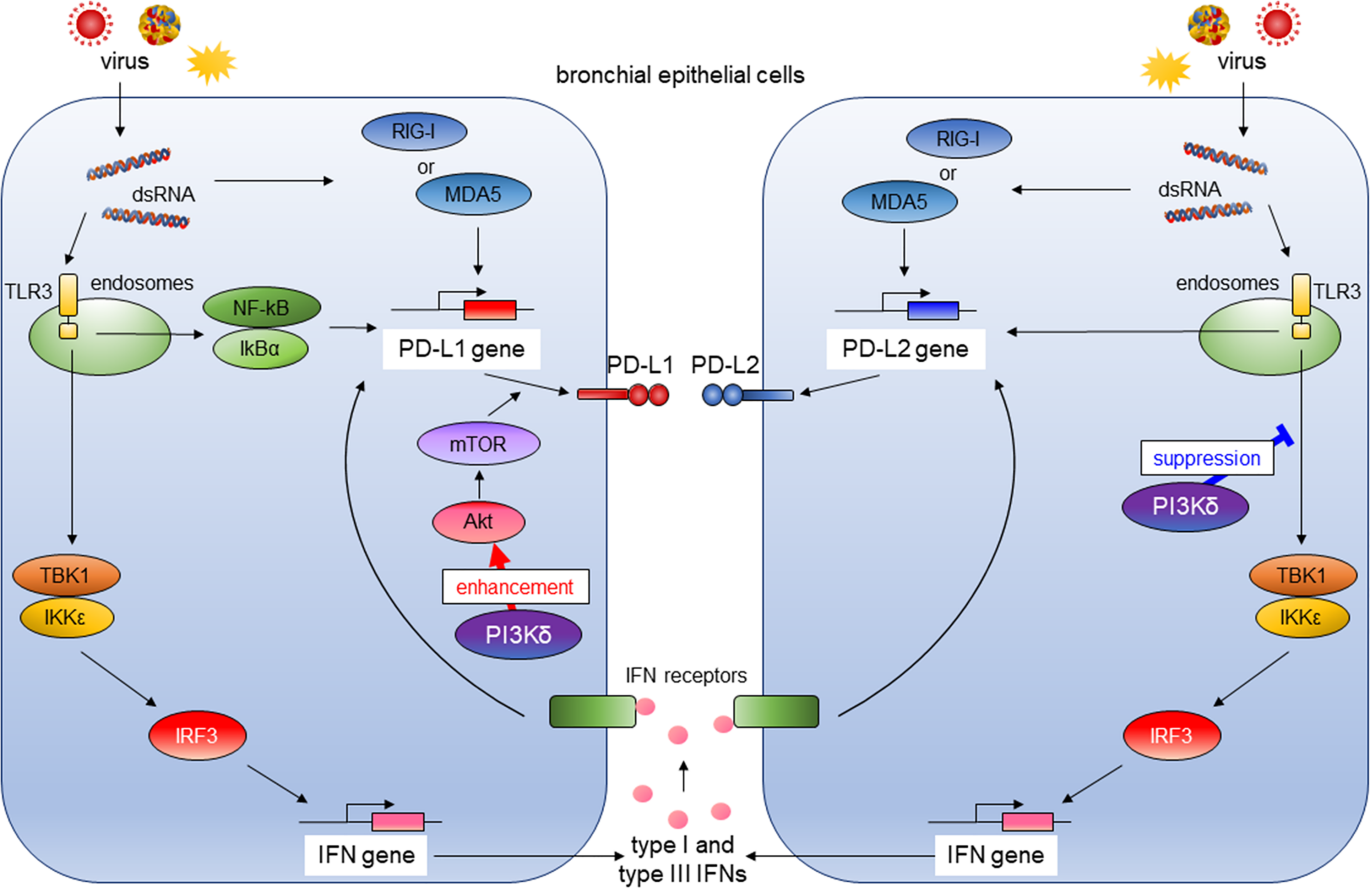
The role of PI3Kδ in antiviral IFN responses and virus-induced expression of PD-L1 and PD-L2 in bronchial epithelial cells. RIG-I, retinoic acid-inducible gene I; MDA5, melanoma Differentiation-Associated protein 5; TLR3, toll-like receptor 3; NF-κB; the nuclear factor kappa B, IκBα; inhibitory kappa Bα, TBK1, the TANK-binding kinase-1; IKKϵ, inhibitor of κB kinase ϵ; IRF3, IFN regulatory factor 3; Akt, v-akt murine thymoma viral oncogene homolog; mTOR, the mechanistic target of rapamycin.

Activation of PI3K pathway is involved in lung tumorigenesis (24). Therefore, the use of lung cancer–derived bronchial epithelial cell lines such as A549 may not be suitable for analyzing involvement of PI3Kδ signaling in antiviral IFN responses and induction of co-inhibitory molecules by virus infection. Hence, we assessed the influence of antiviral IFNs or PI3Kδ on the expressions of PD-L1 and PD-L2 using PBECs. We previously reported that poly I:C induced PD-L1 expression on bronchial epithelial cells *via* the NF-κB pathway (23). Our results showed that exogenous type I and III IFNs also could increase PD-L1 and PD-L2 in PBECs, and IFN combined poly I:C additively enhanced PD-L1 and PD-L2 expressions. The selective PI3Kδ inhibitor IC87114 as well as siRNA-mediated knockdown of the *PIK3CD* gene enhanced poly I:C–induced PD-L2 expression, whereas IC87114 suppressed poly I:C–induced PD-L1 possibly by inhibiting translational induction of PD-L1 *via* the Akt/mTOR pathway. Treatment with IC87114 also enhanced the poly I:C–induced gene expressions of IFNs and IRGs *via* increased phosphorylation of TBK1 and IRF3. siRNA-mediated knockdown of the *IRF3* gene counteracted the enhancement of poly I:C–induced PD-L2 by IC87114, suggesting that PI3Kδ negatively regulates poly I:C–induced IFNs production and subsequent induction of PD-L2 in PBECs (**Figure 9**). We further confirmed similar effects of IC87114 on PD-L1 and PD-L2 expression in PBECs from patients with asthma or COPD and after hMPV infection.

Previous studies showed that type I IFNs increased PD-L1 expression on microvascular endothelial cells, monocytes, and dendritic cells (15, 16). Garcia-Diaz *et al.* reported that IFNβ induced both PD-L1 and PD-L2 expressions in melanoma cells and exerted a stronger effect on PD-L2 expression through its binding of signal transducer and activator of transcription 3 to the PD-L2 promoter (17). Here we showed for the first time that antiviral type I and III IFNs induced PD-L1 and PD-L2 in PBECs, and this induction was increased by co-stimulation with IFNs and the viral dsRNA analog. Similar to our observations, Hastings *et al.* also reported that hMPV infection in type I IFN receptor–deficient mice induced lower PD-L1 expression on lung epithelial cells compared with levels in wild-type mice (25). The recognition of virus-associated molecules by host PRRs after viral infection mediates the induction of the innate immune response typified by the production of antiviral IFNs and IRGs and the subsequent development of adaptive immunity to viruses (26, 27). Research has shown that the PD-1/PD-L1 axis is responsible for CD8^+^ T cell exhaustion and failure to achieve viral clearance in acute respiratory virus infections (4, 5). However, the role of PD-L2 in adaptive immunity has not been fully elucidated. PD-L2 binds not only to PD-1 but also repulsive guidance molecule b (RGMb), and the PD-L2/RGMb interaction promotes CD4^+^ T-helper cell-1 (Th1) responses (28, 29). In malarial infections, PD-L2 expression on dendritic cells inversely correlated with disease severity, and PD-L2 improved parasite-specific CD4^+^ Th1 responses and inhibited PD-L1 binding to PD-1 (30). These studies demonstrate the differential co-inhibitory function of PD-L1 and co-stimulatory function of PD-L2 in T-cell immunity.

Viral infections of the airway are one of the leading causes of asthma and COPD exacerbation and accelerate disease progression (31, 32). Therefore, antiviral strategies targeting the underlying pathogenesis of asthma and COPD are needed. We previously showed that the NF-κB pathway plays an essential role in poly I:C–induced upregulation of PD-L1, and IC87114 attenuated poly I:C–induced PD-L1 expression in healthy PBECs by inhibiting translational induction of PD-L1 *via* the Akt/mTOR pathway (14, 23). In this study, we observed suppressive effects of IC87114 on poly I:C–induced PD-L1 in PBECs from asthma patients, but not patients with COPD. Chronic exposure to cigarette smoke is a major etiologic factor in the pathogenesis of COPD and is reported to induce PD-L1 expression in human bronchial epithelial cells and murine lungs through the aryl hydrocarbon receptor (33). This etiology-specific mechanism underlying the upregulation of PD-L1 expression may be the reason for the insufficient suppressive effect of IC87114 on poly I:C–induced PD-L1 in PBECs from patients with COPD. Contrary to IC87114-induced suppression of PD-L1 expression, IC87114 enhanced poly I:C– or hMPV-induced PD-L2 gene and protein expressions in PBECs, possibly *via* increased production of antiviral IFNs (**Figure 9**). Our findings, along with the previous studies of the role of PD-L1 and PD-L2 expressions in T-cell immunity, suggest that a selective PI3Kδ inhibitor may simultaneously enhance innate antiviral and adaptive immunity and represent an efficacious treatment for respiratory virus infections.

One limitation of our study was that we did not analyze the effect of IC87114 on adaptive immune responses in other cells. Further *in vivo* investigations are needed to evaluate whether a PI3Kδ inhibitor activates T-cell immunity through changes in PD-L1 and PD-L2 expression in the lungs and promotes viral clearance from the infected lungs. Another limitation was that we analyzed the effects of IC87114 on PD-L1 and PD-L2 expression using PBECs from patients with relatively mild asthma and COPD. It remains unclear whether similar effects are observed in patients with severe asthma and COPD.

In conclusion, we showed that antiviral IFNs and poly I:C additively induced PD-L1 and PD-L2 expressions in PBECs. The selective PI3Kδ inhibitor IC87114 differentially regulated poly I:C–and virus-induced PD-L1 and PD-L2 expressions in healthy PBECs as well as PBECs from asthma patients and COPD patients. IC87114 further increased poly I:C–induced PD-L2 expression possibly *via* enhanced induction of antiviral IFNs. The use of a selective PI3Kδ inhibitor may be beneficial for the treatment of respiratory viral infections by enhancing the crosstalk between innate and adaptive immune responses.

## Data Availability Statement

The original contributions presented in the study are included in the article/**Supplementary Material**. Further inquiries can be directed to the corresponding author.

## Ethics Statement

The studies involving human participants were reviewed and approved by the Kyushu University Institutional Review Board for Clinical Research. The patients/participants provided their written informed consent to participate in this study.

## Author Contributions

KK, YI, SF, and KM conceived the study design and supervised the scientific work. TO, AS, and AF performed the experiments. TO and KK analyzed the data. All authors contributed to and approved the final manuscript.

## Funding

This work was supported by JSPS KAKENHI Grant Number JP18K15953, JP19K08626 and JP21K15471, and the Centre for Clinical and Translational Research of Kyushu University.

## Conflict of Interest

KM received an honorarium for educational lectures from AstraZeneca and GlaxoSmithKline Pharmaceuticals Ltd.

The remaining authors declare that the research was conducted in the absence of any commercial or financial relationships that could be construed as a potential conflict of interest.

## Publisher’s Note

All claims expressed in this article are solely those of the authors and do not necessarily represent those of their affiliated organizations, or those of the publisher, the editors and the reviewers. Any product that may be evaluated in this article, or claim that may be made by its manufacturer, is not guaranteed or endorsed by the publisher.

## References

[1] Pauken KE Wherry EJ. Overcoming T Cell Exhaustion in Infection and Cancer. Trends Immunol (2015) 36:265–76. doi: 10.1016/j.it.2015.02.008 PMC4393798 25797516

[2] Rogers MC Williams JV. Reining in the CD8+ T Cell: Respiratory Virus Infection and PD-1-Mediated T-Cell Impairment. PloS Pathog (2019) 15:e1007387. doi: 10.1371/journal.ppat.1007387 30605483 PMC6317792

[3] Schonrich G Raftery MJ. The PD-1/PD-L1 Axis and Virus Infections: A Delicate Balance. Front Cell Infect Microbiol (2019) 9:207. doi: 10.3389/fcimb.2019.00207 31263684 PMC6584848

[4] Erickson JJ Gilchuk P Hastings AK Tollefson SJ Johnson M Downing MB. Viral Acute Lower Respiratory Infections Impair CD8+ T Cells Through PD-1. J Clin Invest (2012) 122:2967–82. doi: 10.1172/JCI62860 PMC3408742 22797302

[5] Mcnally B Ye F Willette M Flano E. Local Blockade of Epithelial PDL-1 in the Airways Enhances T Cell Function and Viral Clearance During Influenza Virus Infection. J Virol (2013) 87:12916–24. doi: 10.1128/JVI.02423-13 PMC3838157 24067957

[6] Stanciu LA Bellettato CM Laza-Stanca V Coyle AJ Papi A Johnston SL. Expression of Programmed Death-1 Ligand (PD-L) 1, PD-L2, B7-H3, and Inducible Costimulator Ligand on Human Respiratory Tract Epithelial Cells and Regulation by Respiratory Syncytial Virus and Type 1 and 2 Cytokines. J Infect Dis (2006) 193:404–12. doi: 10.1086/499275 16388488

[7] Alexopoulou L Holt AC Medzhitov R Flavell RA. Recognition of Double-Stranded RNA and Activation of NF-Kappab by Toll-Like Receptor 3. Nature (2001) 413:732–8. doi: 10.1038/35099560 11607032

[8] Yoneyama M Kikuchi M Natsukawa T Shinobu N Imaizumi T Miyagishi M. The RNA Helicase RIG-I has an Essential Function in Double-Stranded RNA-Induced Innate Antiviral Responses. Nat Immunol (2004) 5:730–7. doi: 10.1038/ni1087 15208624

[9] Kato H Takeuchi O Sato S Yoneyama M Yamamoto M Matsui K. Differential Roles of MDA5 and RIG-I Helicases in the Recognition of RNA Viruses. Nature (2006) 441:101–5. doi: 10.1038/nature04734 16625202

[10] Levy DE Marie IJ Durbin JE. Induction and Function of Type I and III Interferon in Response to Viral Infection. Curr Opin Virol (2011) 1:476–86. doi: 10.1016/j.coviro.2011.11.001 PMC3272644 22323926

[11] Zhou JH Wang YN Chang QY Ma P Hu Y Cao X. Type III Interferons in Viral Infection and Antiviral Immunity. Cell Physiol Biochem (2018) 51:173–85. doi: 10.1159/000495172 30439714

[12] Tsuda M Matsumoto K Inoue H Matsumura M Nakano T Mori A. Expression of B7-H1 and B7-DC on the Airway Epithelium is Enhanced by Double-Stranded RNA. Biochem Biophys Res Commun (2005) 330:263–70. doi: 10.1016/j.bbrc.2005.02.161 15781259

[13] Kan-O K Matsumoto K Asai-Tajiri Y Fukuyama S Hamano S Seki N. PI3K-Delta Mediates Double-Stranded RNA-Induced Upregulation of B7-H1 in BEAS-2B Airway Epithelial Cells. Biochem Biophys Res Commun (2013) 435:195–201. doi: 10.1016/j.bbrc.2013.04.082 23660190

[14] Fujita A Kan-O K Tonai K Yamamoto N Ogawa T Fukuyama S. Inhibition of PI3Kdelta Enhances Poly I:C-Induced Antiviral Responses and Inhibits Replication of Human Metapneumovirus in Murine Lungs and Human Bronchial Epithelial Cells. Front Immunol (2020) 11:432. doi: 10.3389/fimmu.2020.00432 32218789 PMC7079687

[15] Eppihimer MJ Gunn J Freeman GJ Greenfield EA Chernova T Erickson J. Expression and Regulation of the PD-L1 Immunoinhibitory Molecule on Microvascular Endothelial Cells. Microcirculation (2002) 9:133–45. doi: 10.1038/sj/mn/7800123 PMC3740166 11932780

[16] Schreiner B Mitsdoerffer M Kieseier BC Chen L Hartung HP Weller M. Interferon-Beta Enhances Monocyte and Dendritic Cell Expression of B7-H1 (PD-L1), a Strong Inhibitor of Autologous T-Cell Activation: Relevance for the Immune Modulatory Effect in Multiple Sclerosis. J Neuroimmunol (2004) 155:172–82. doi: 10.1016/j.jneuroim.2004.06.013 15342209

[17] Garcia-Diaz A Shin DS Moreno BH Saco J Escuin-Ordinas H Rodriguez GA. Interferon Receptor Signaling Pathways Regulating PD-L1 and PD-L2 Expression. Cell Rep (2017) 19:1189–201. doi: 10.1016/j.celrep.2017.04.031 PMC6420824 28494868

[18] Okkenhaug K. Signaling by the Phosphoinositide 3-Kinase Family in Immune Cells. Annu Rev Immunol (2013) 31:675–704. doi: 10.1146/annurev-immunol-032712-095946 23330955 PMC4516760

[19] Kan-O K Ramirez R Macdonald MI Rolph M Rudd PA Spann KM. Human Metapneumovirus Infection in Chronic Obstructive Pulmonary Disease: Impact of Glucocorticosteroids and Interferon. J Infect Dis (2017) 215:1536–45. doi: 10.1093/infdis/jix167 28379462

[20] Foster MW Gerhardt G Robitaille L Plante PL Boivin G Corbeil J. Targeted Proteomics of Human Metapneumovirus in Clinical Samples and Viral Cultures. Anal Chem (2015) 87:10247–54. doi: 10.1021/acs.analchem.5b01544 26376123

[21] Garcia CC Tavares LP Dias ACF Kehdy F Alvarado-Arnez LE Queiroz-Junior CM. Phosphatidyl Inositol 3 Kinase-Gamma Balances Antiviral and Inflammatory Responses During Influenza a H1N1 Infection: From Murine Model to Genetic Association in Patients. Front Immunol (2018) 9:975. doi: 10.3389/fimmu.2018.00975 29867955 PMC5962662

[22] Fitzgerald KA Mcwhirter SM Faia KL Rowe DC Latz E Golenbock DT. Ikkepsilon and TBK1 are Essential Components of the IRF3 Signaling Pathway. Nat Immunol (2003) 4:491–6. doi: 10.1038/ni921 12692549

[23] Kan-O K Matsumoto K Inoue H Fukuyama S Asai Y Watanabe W. Corticosteroids Plus Long-Acting Beta2-Agonists Prevent Double-Stranded RNA-Induced Upregulation of B7-H1 on Airway Epithelium. Int Arch Allergy Immunol (2013) 160:27–36. doi: 10.1159/000338430 22948082

[24] Fumarola C Bonelli MA Petronini PG Alfieri RR. Targeting PI3K/AKT/Mtor Pathway in non Small Cell Lung Cancer. Biochem Pharmacol (2014) 90:197–207. doi: 10.1016/j.bcp.2014.05.011 24863259

[25] Hastings AK Erickson JJ Schuster JE Boyd KL Tollefson SJ Johnson M. Role of Type I Interferon Signaling in Human Metapneumovirus Pathogenesis and Control of Viral Replication. J Virol (2015) 89:4405–20. doi: 10.1128/JVI.03275-14 PMC4442394 25653440

[26] Kawai T Akira S. Innate Immune Recognition of Viral Infection. Nat Immunol (2006) 7:131–7. doi: 10.1038/ni1303 16424890

[27] Sadler AJ Williams BR. Interferon-Inducible Antiviral Effectors. Nat Rev Immunol (2008) 8:559–68. doi: 10.1038/nri2314 PMC2522268 18575461

[28] Xiao Y Yu S Zhu B Bedoret D Bu X Francisco LM. Rgmb is a Novel Binding Partner for PD-L2 and its Engagement With PD-L2 Promotes Respiratory Tolerance. J Exp Med (2014) 211:943–59. doi: 10.1084/jem.20130790 PMC4010901 24752301

[29] Nie X Chen W Zhu Y Huang B Yu W Wu Z. B7-DC (PD-L2) Costimulation of CD4(+) T-Helper 1 Response via Rgmb. Cell Mol Immunol (2018) 15:888–97. doi: 10.1038/cmi.2017.17 PMC6207567 28479601

[30] Karunarathne DS Horne-Debets JM Huang JX Faleiro R Leow CY Amante F. Programmed Death-1 Ligand 2-Mediated Regulation of the PD-L1 to PD-1 Axis is Essential for Establishing CD4(+) T Cell Immunity. Immunity (2016) 45:333–45. doi: 10.1016/j.immuni.2016.07.017 27533014

[31] Jackson DJ Sykes A Mallia P Johnston SL. Asthma Exacerbations: Origin, Effect, and Prevention. J Allergy Clin Immunol (2011) 128:1165–74. doi: 10.1016/j.jaci.2011.10.024 PMC7172902 22133317

[32] Linden D Guo-Parke H Coyle PV Fairley D Mcauley DF Taggart CC. Respiratory Viral Infection: A Potential “Missing Link” in the Pathogenesis of COPD. Eur Respir Rev (2019) 28. doi: 10.1183/16000617.0063-2018 PMC9488189 30872396

[33] Wang GZ Zhang L Zhao XC Gao SH Qu LW Yu H. The Aryl Hydrocarbon Receptor Mediates Tobacco-Induced PD-L1 Expression and is Associated With Response to Immunotherapy. Nat Commun (2019) 10:1125. doi: 10.1038/s41467-019-08887-7 30850589 PMC6408580

