# Supplementary material for: Inhibition of PI3Kδ Differentially Regulates Poly I:C– and Human Metapneumovirus–Induced PD–L1 and PD–L2 Expression in Human Bronchial Epithelial Cells
Source: Front Immunol. 2021 Nov 25;12:767666. doi: 10.3389/fimmu.2021.767666 (PMC8656419; doi:10.3389/fimmu.2021.767666)
Supplement: Supplementary file 1 [file DataSheet_1.pdf]

Table S1: Demographic data of patients with asthma and COPD

	Asthma<br>n=3	COPD<br>n=5
Age, years	68/69/56	73/64/73/77/72
Gender	Female/Female/Female	Male/Female/Male/Male/Male
Smoking history, pack-years	0/0/0	30/42/40/29/40
Blood eosinophil counts, /μL	380/292/580	76/203/240/179/380
FEV_1 % predicted (post-BD), %	66/80/109	72/116/97/72/32
FEV_1/FVC (post-BD), %	59/71/79	50/66/60/64/40
BD response (% increase in FEV_1), %	20/7/ND	3/2/5/0/ND
CT scan findings of emphysema	−/−/−	+/+/+/+/+
Treatment	SABA*/ICS＋LABA,<br>SABA*/ ICS＋LABA, SABA*	LAMA+LABA/LAMA+LABA/−/<br>LAMA+LABA/−

BD, bronchodilator; ND, not done, SABA, short-acting β_2-agonist; ICS, inhaled corticosteroids; LABA, long-acting β_2-agonist; LAMA, long-acting muscarinic antagonist. *Use of as-needed medication

Table S2: Primer sequences used for qRT-PCR in this study.

IFNB1	Sense: 5′ TTGACATCCCTGAGGAGATTAAGC 3′
	Antisense: 5′ TTAGCCAGGAGGTTCTCAACAATAG 3′
IFNL1	Sense: 5′ CTAGACCAGCCCCTTCACAC 3′
	Antisense: 5′ AAGGTGACAGATGCCTCCAG 3′
MxA	Sense: 5′ GTGCATTGCAGAAGGTCAGA 3′
	Antisense: 5′ TTCAGGAGCCAGCTGTAGGT 3′
ISG56	Sense: 5′ GCAGCCAAGTTTTACCGAAG 3′
	Antisense: 5′ CACCTCAAATGTGGGCTTTT 3′
CD274	Sense: 5′ CAATGTGACCAGCACACTGAGAA 3′
	Antisense: 5′ GGCATAATAAGATGGCTCCCAGAA 3′
CD273	Sense: 5′ AAAGACCTGTCACCACAACAAAG 3′
	Antisense: 5′ AAAGTGCTGGGTCATCCAAAG 3′

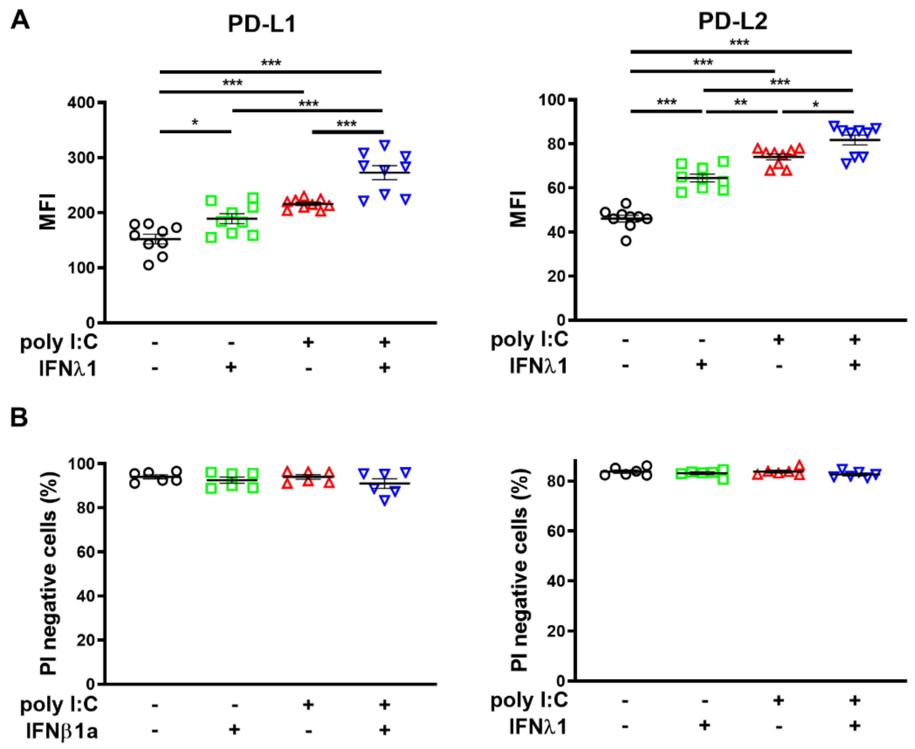

Figure S1. Antiviral interferon increases the expression of PD-L1 and PD-L2 on PBECs; Related to Figure 1.

PBECs were pretreated with 250 U/ml IFNλ1 **(A, B)**, 250 U/ml IFNβ1a **(B)**, or vehicle for 1 h and then stimulated with 1 μg/mL poly I:C for 24 h. PD-L1 expression, PD-L2 expression, or PI-negative viable cells were analyzed using flow cytometry. Data represent means ± SEM (n=6-9 per group) and were pooled from a minimum of two independent donors with three replicates. **p*<0.05, ***p*<0.01, ****p*<0.001 by one-way ANOVA. MFI, mean fluorescence intensity.

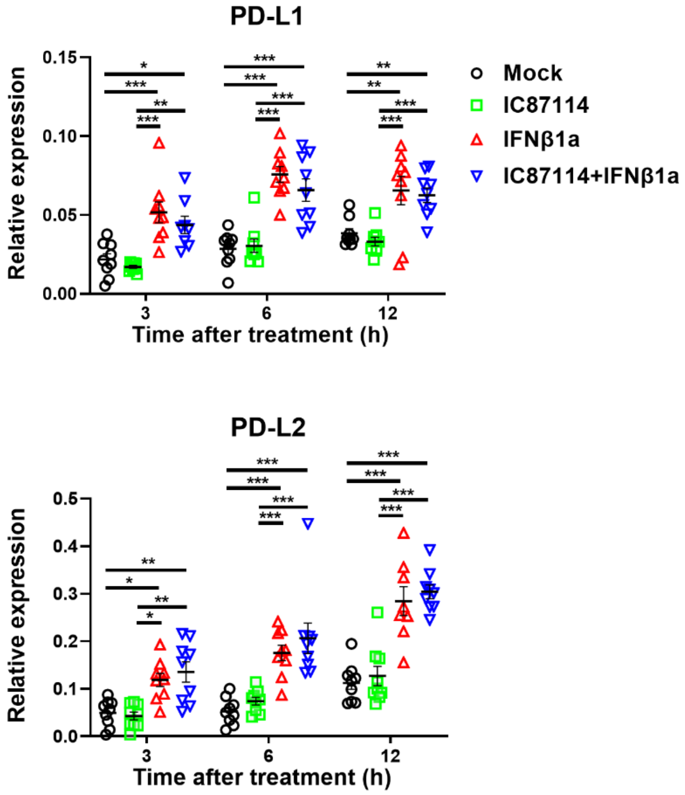

Figure S2. Effects of IC87114 on IFNβ–induced gene expression of PD-L1 and PD-L2 in PBECs; Related to Figure 3.

PBECs were treated with 10nM IC87114 and/or 250 U/ml IFNβ1a, or vehicle and then PD-L1 and PD-L2 gene expressions were measured at the indicated times by real-time quantitative reverse-transcription PCR and normalized to that of 18S rRNA. Data represent means ± SEM (n=9 per group) and pooled from three independent donors with three replicates. *p<0.05, **p<0.01, ***p<0.001 by two-way ANOVA.

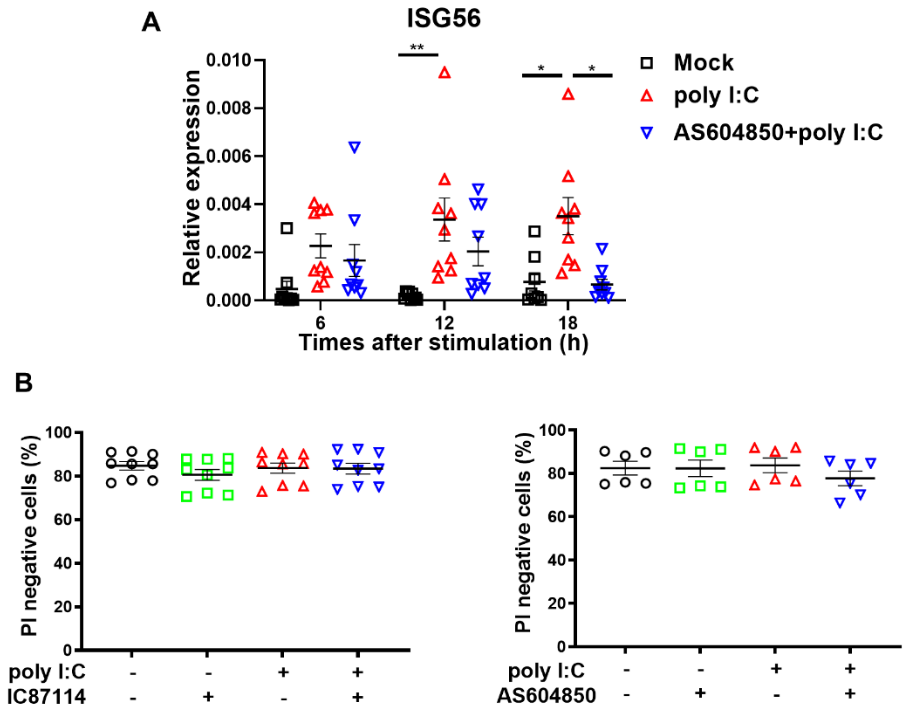

Figure S3. A PI3Kγ inhibitor suppresses poly I:C–induced gene expression of *ISG56* in PBECs; Related to Figure 3.

PBECs were pretreated with10 μM the PI3Kγ inhibitor AS604850 **(A, B)**, 10 μM the PI3Kδ inhibitor IC87114 **(B)**, or vehicle for 1 h and then stimulated with 1 μg/mL poly I:C. **(A)** ISG56 gene expressions were measured at the indicated times by real-time quantitative reverse-transcription PCR and normalized to that of 18S rRNA. **(B)** PI-negative viable cells were analyzed 24 h following stimulation using flow cytometry. Data represent means ± SEM (n=6-9 per group) and pooled from a minimum of two independent donors with three replicates. *p<0.01, **p<0.001 by one- or two-way ANOVA as appropriate.

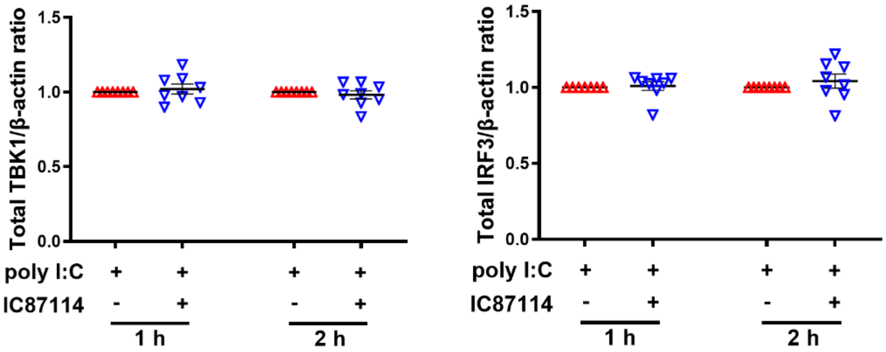

Figure S4. Effects of IC87114 on protein levels of total TBK1 and IRF3 in PBECs stimulated with poly I:C; Related to Figure 5.

PBECs were pretreated with 10nM IC87114 or vehicle for 1 h, then stimulated with 1 μg/mL poly I:C. Cells were collected 1 h and 2 h following stimulation, then protein levels of total TBK1 or IRF3 were analyzed by western blotting. Band intensity was quantitated using densitometry. Data represent means ± SEM (n=8 per group) and pooled from four independent donors with two replicates. Differences in data were analyzed by one-way ANOVA.

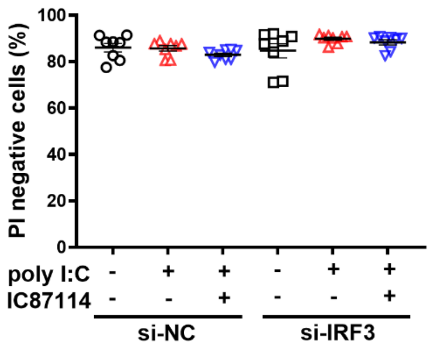

Figure S5. Effects of siRNA knockdown of the *IRF3* gene and stimulation with poly I:C with or without IC87114 treatment on cell viability; Related to Figure 6.

PBECs were transfected with IRF3 siRNA or NC siRNA for 48 h, then cells were pretreated with 10nM IC87114 or vehicle for 1 h followed by stimulation with 1 μg/mL poly I:C. Viable cells (PI negative) were identified 24 h following stimulation using flow cytometry. Data represent means ± SEM (n=8-9 per group) and pooled from three independent donors with three replicates. Differences in data were analyzed by one-way ANOVA.

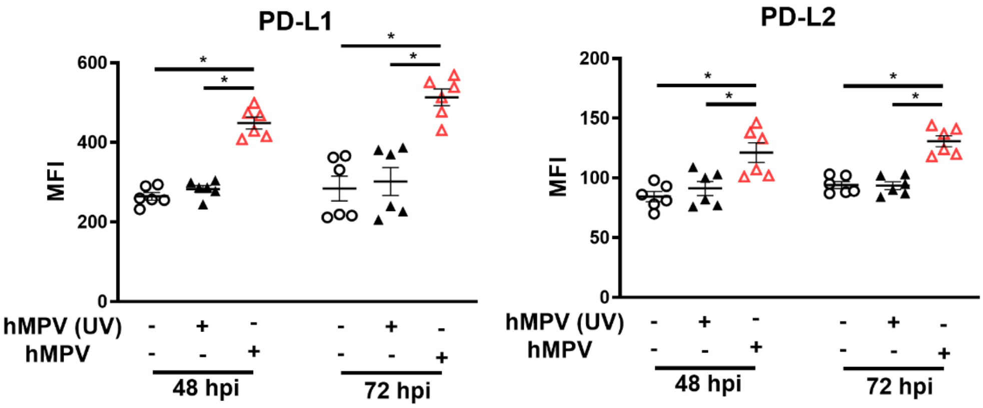

Figure S6. UV-irradiated hMPV was unable to induce PD-L1 or PD-L2 expression on PBECs; Related to Figure 8. PBECs were infected with hMPV (MOI 0.1) or UV-irradiated hMPV (MOI 0.1). PD-L1 and PD-L2 expression was analyzed at 48 and 72 h post-infection using flow cytometry. Data represent means ± SEM (n=6 per group) and pooled from two independent donors with three replicates. *$p<0.001$ by two-way ANOVA. MFI, mean fluorescence intensity.
